# Daphnetin: A Novel Anti-*Helicobacter pylori* Agent

**DOI:** 10.3390/ijms20040850

**Published:** 2019-02-15

**Authors:** Genzhu Wang, Jing Pang, Xinxin Hu, Tongying Nie, Xi Lu, Xue Li, Xiukun Wang, Yun Lu, Xinyi Yang, Jiandong Jiang, Congran Li, Yan Q Xiong, Xuefu You

**Affiliations:** 1 Beijing Key Laboratory of Antimicrobial Agents, Institute of Medicinal Biotechnology, Chinese Academy of Medical Sciences and Peking Union Medical College, Beijing 100050, China; wanggenzhu_890813@163.com (G.W.); pangjing.pangjing@163.com (J.P.); huxinxin1985@163.com (X.H.); 15010375866@163.com (T.N.); luxi23@126.com (X.L.); xli8891@163.com (X.L.); xiukunwang@139.com (X.W.); yunluly@163.com (Y.L.); yangxinyi1976@hotmail.com (X.Y.); jiang.jdong@163.com (J.J.); 2 Los Angeles Biomedical Research Institute, Harbor-UCLA Medical Center, Torrance, CA 90502, USA; 3 David Geffen School of Medicine at UCLA, Los Angeles, CA 90095, USA

**Keywords:** *Helicobacter pylori*, daphnetin, mechanism of action, colonization

## Abstract

Background: Antibiotic-resistant *H. pylori* was increasingly found in infected individuals, which resulted in treatment failure and required alternative therapeutic strategies. Daphnetin, a coumarin-derivative compound, has multiple pharmacological activities. Methods: The mechanism of daphnetin on *H. pylori* was investigated focusing on its effect on cell morphologies, transcription of genes related to virulence, adhesion, and cytotoxicity to human gastric epithelial (GES-1) cell line. Results: Daphnetin showed good activities against multidrug resistant (MDR) *H. pylori* clinical isolates, with minimal inhibitory concentration (MIC) values ranging from 25 to 100 μg/mL. In addition, daphnetin exposure resulted in *H. pylori* morphological changes. Moreover, daphnetin caused increased translocation of phosphatidylserine (PS), DNA damage, and *recA* expression, and RecA protein production vs. control group. Of great importance, daphnetin significantly decreased *H. pylori* adhesion to GES-1 cell line vs. control group, which may be related to the reduced expression of colonization related genes (e.g., *babA* and *ureI*). Conclusions: These results suggested that daphnetin has good activity against MDR *H. pylori.* The mechanism(s) of daphnetin against *H. pylori* were related to change of membrane structure, increase of DNA damage and PS translocation, and decrease of *H. pylori* attachment to GES-1 cells.

## 1. Introduction

*Helicobacter pylori (H. pylori)*, a Gram-negative bacterium that specifically colonizes in the human stomach, has developed numerous strategies to survive in the high acidity environment in the stomach lumen [1]. It has been reported that this pathogen chronically infects over half of all humans [2]. Colonization of *H. pylori* can lead to gastritis and peptic ulcers, mucosa-associated lymphoid tissue lymphoma, and gastric cancer [3]. Therefore, *H. pylori* has been categorized as a Class I carcinogen by the World Health Organization (WHO) [4]. In addition, *H. pylori* infections are more common in developing countries and are mostly developed during childhood [5]. Of note, anti-*H. pylori* therapy has been used for decades, but the efficacy of the treatment has declined during the last decade because of increasing antibiotic resistance [6,7]. In 2017, WHO listed 12 bacteria that threaten human health the greatest, among which clarithromycin-resistant *H. pylori* was considered to be one of the high priorities [8]. A recent review demonstrated that *H. pylori* resistance rate to clarithromycin was 28.9%, whereas the primary antibiotic resistance of *H. pylori* was metronidazole in China (around 70%) [9]. Therefore, new antibacterial agents against *H. pylori* are needed to overcome this concern.

**Figure 1:**
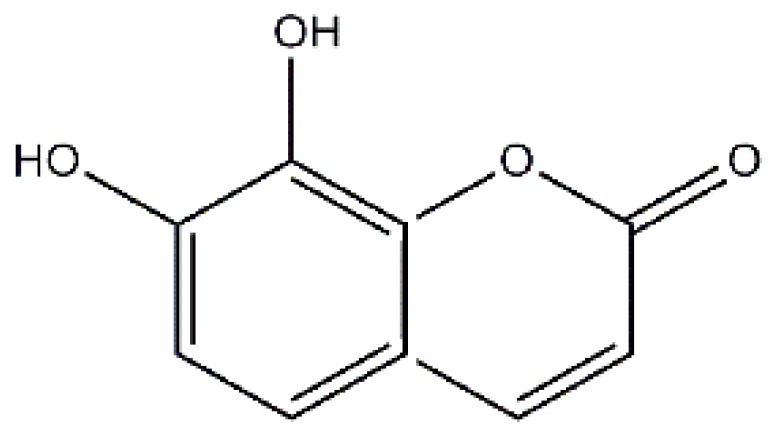
Structure of daphnetin (Molecular Weight 178.143 g/mol).

Daphnetin (7,8-dihydroxycoumarin)—a major bioactive component extracted from the genus *Daphne* as well as several other genera—is a coumarin-derivative compound of aromatic odor, with structure comprising o-hydroxy cinamic acid lactones (Figure 1) [10]. In China, daphnetin has been used clinically to treat Buerger’s disease for many years [11]. Its multiple pharmacological activities, including anti-inflammatory, -diarrheal, -parasitic, -hypoxia, etc., have been reported [12,13]. Daphnetin exhibited selective cytotoxicity to human renal cell carcinoma cells, relative to noncarcinoma proximal tubular cells [14]. So far, neither toxic effects [15] nor genetic toxicity [15,16] were found in daphnetin. Therefore, it has attracted extensive research interests to investigate the activity and mechanism(s) of daphnetin against MDR *H. pylori*.

In the current studies, we examined the antibacterial activity of daphnetin against 20 *H. pylori* clinical isolates, including MDR strains, and investigated its anti-*H. pylori* mechanisms. Our findings suggest that daphnetin may offer a significant advantage in the prevention of *H. pylori* infections.

## 2. Results

### 2.1. Antibacterial Activity of Daphnetin Against H. pylori Strains

A total of 20 *H. pylori* strains isolated from human gastric antrum were used in this study (Table S1). Daphnetin inhibited all the tested *H. pylori* strains, regardless of their resistance profiles to other common used antibiotics, at concentrations ranging from 25 to 100 μg/mL (Table 1 and Table S1). The percentage of clarithromycin resistance in the studied *H. pylori* strains was 25%, with MICs ranging from 0.016 to 4 μg/mL (Table 1). Eighty-five percent of the clinical *H. pylori* strains were resistant to metronidazole with MICs ranging from 4 to 256 μg/mL, and daphnetin still had good activity against these highly metronidazole resistant *H. pylori* strains with MICs of 25 μg/mL (Table 1 and Table S1). *H. pylori* ATCC43504 strain was used as a quality control with expected MICs of clarithromycin and metronidazole (Table 1).

### 2.2. Effect of Daphnetin on H. pylori Morphology

**Figure 2:**
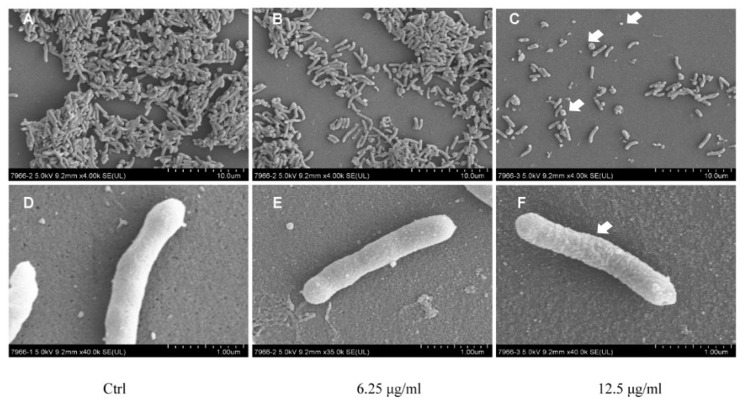
The morphology of *H. pylori* cells with/without daphnetin exposure observed by SEM. Control (**A**,**D**); *H. pylori* were treated with 6.25 μg/mL (**B**,**E**) or 12.5 μg/mL of daphnetin (**C**,**F**). Magnification: A–C = 4000; D–F = 40000.

**Figure 3:**
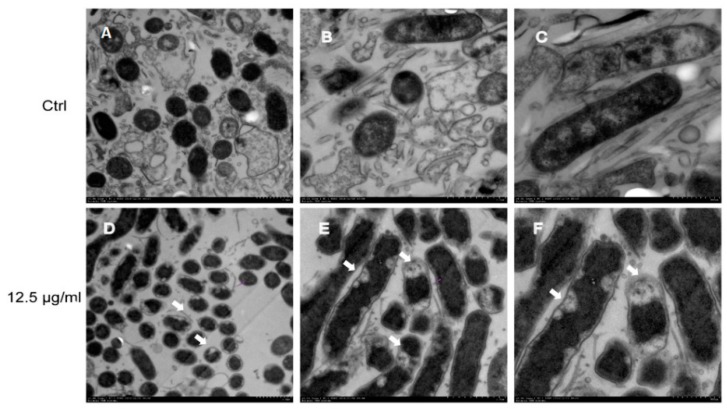
The morphology of *H. pylori* cells with/without daphnetin exposure observed by TEM. Control (**A**–**C**); *H. pylori* treated with 12.5 μg/mL daphnetin (**D**–**F**). Magnification: A and D = 3000; B and E = 5000; and C and F = 8000.

We first visualized the morphology of entire *H. pylori* cells by scanning electron microscopy (SEM). Control cells without daphnetin exposure demonstrated smooth, homogenous cell surfaces and rod-shaped morphotypes (Figure 2A,D). After exposure to daphnetin at sub-MIC concentrations (e.g., 6.25 or 12.5 μg/mL) for three days, some *H. pylori* cells showed extensive surface damage (e.g., budding structures), heterogeneous populations of cells, and increased coccid forms in a concentration-dependent manner (Figure 2B,E for daphnetin at 6.25 μg/mL exposure; 2C,F for daphnetin at 12.5 μg/mL exposure). Transmission electron microscopy (TEM) images displayed the organization of *H. pylori* with a clearly defined cytoplasm and cell membrane. The ultrastructural characteristics of *H. pylori* cells without daphnetin exposure showed homogeneous cytoplasm and intact cell membrane (Figure 3A–C). However, daphnetin-treated (at 12.5 μg/mL) *H. pylori* cells showed visual morphological changes, including reduced bacterial size (relative diameter: control:daphnetin exposure group = 1.00:0.77), rough outer membrane, granular-textured cytoplasm, peculiar detachments between membrane and cytoplasm, numerous vesicles, and/or typical “holes” attached to the inner membrane. In addition, vesicles emerged in ~40% *H. pylori* cells in the daphnetin exposure group, while no vesicle was found in control group (Figure 3D–F). Taken together, electron microscopy images showed that daphnetin exposed *H. pylori* had substantial visual morphological changes.

### 2.3. Daphnetin-Induced Membrane Changes

The extent of phosphatidylserine (PS) exposure on the outer membrane was determined using annexin V. We observed that daphnetin at sub-MIC concentration (12.5 μg/mL) resulted in significant increased annexin V-mediated labeling of PS, from 5.93% to 56.99% (Table 2). These data indicated that daphnetin could change *H. pylori’*s outer membrane structure. We also employed the bis-(1,3-dibutylbarbituric acid) trimethine oxonol (DiBAC) and propidium iodide (PI) dye to monitor membrane polarity and permeability. As shown in Table 2, no significant fluorescence changes were observed between control and daphnetin-treated *H. pylori* cells. Subsequently, we demonstrated that protein leakage was slightly increased in daphnetin treated group vs. control group (Table 2). However, these differences did not reach statistical significance. These results suggested that daphnetin treatment led to outer membrane structure change, while had no significant effect on membrane permeability and depolarization in *H. pylori.*

### 2.4. Daphnetin Caused DNA Damage

**Figure 4:**
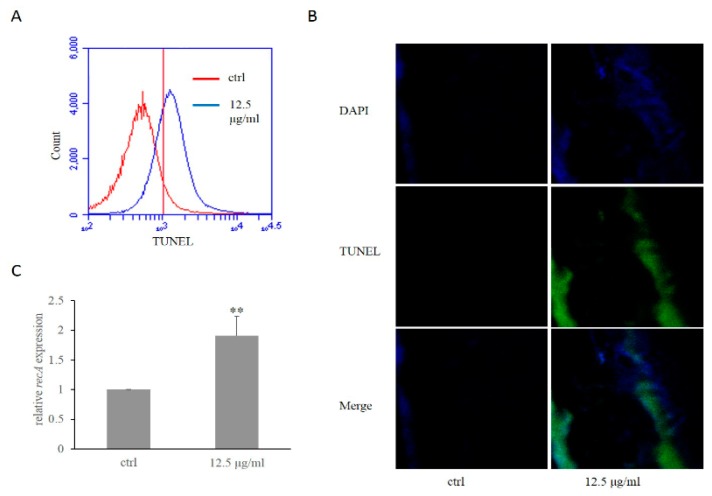
Detection of DNA damage and *recA* expression in *H. pylori*. DNA damage detected using TUNEL by flow cytometry (**A**) and confocal (**B**). (**C**) The expression of *recA* in *H. pylori* with/without daphnetin exposure. (** *p* < 0.001 vs. control). The expression of the study genes without daphnetin exposure was normalized as 1.

In order to determine whether daphnetin was able to cause DNA damage in *H. pylori*, flow cytometry and confocal were used to detect terminal deoxynucleotidyl transferase dUTP nick-end labeling (TUNEL) staining cells. As shown in Figure 4A, significant higher fluorescence signal was observed in daphnetin-treated *H. pylori* cells vs. control (8.11% vs. 68.02% for control and daphnetin- treated group, respectively). These results were confirmed with confocal analyses (Figure 4B). It is well known that RecA is linked between DNA damage and membrane structure changes [17]. Thus, we tested the transcription level of *recA* and found that *recA* expression was significantly increased by daphnetin exposure as compared to untreated cells (Figure 4C). Corresponding to *recA* expression results, RecA protein production was also significantly increased in the daphnetin exposure group vs. control (66.9 ± 6.1 to 133.1 ± 6.1 for control and daphnetin-treated group, respectively; *p* < 0.001, Figure S1).

### 2.5. Daphnetin Decreased H. pylori Adherence to Immortalized Human Gastric Epithelial Cell Line (GES-1) and Inhibited Colonization-Associated Gene Expression

**Figure 5:**
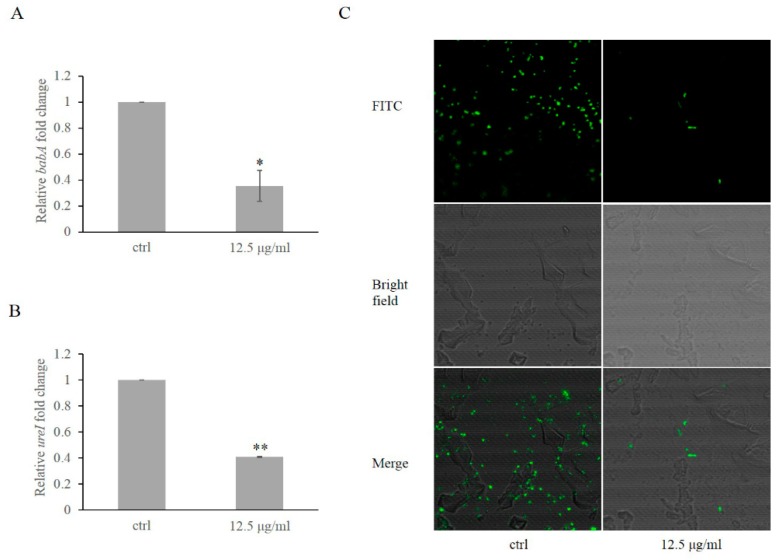
The transcription of *babA* (**A**) and *ureI* (**B**) in *H. pylori* with/without daphnetin exposure. The expression of the study genes without daphnetin exposure was normalized as 1. Inhibitory effect of daphnetin on adhesion of *H. pylori* to GES-1 cells (**C**). The level of adherence of *H. pylori* was detected by confocal (magnification: 600). All the data were presented as mean and standard deviations (SD). * *p* < 0.05, ** *p* < 0.01 vs. control.

In order to investigate the effect of daphnetin on colonization, the expression of two key genes (*babA* and *ureI*) related to colonization were measured in *H. pylori* by qRT-PCR. The transcriptions of the two genes were significantly repressed by daphnetin exposure as compared to untreated cells (Figure 5A,B). In addition, we tested the production of BabA and UreI protein by LC–MS/MS, and found that consistent to the gene expression data, BabA protein level was also decreased in the daphnetin exposure group (from 113.9 ± 10.8 to 86.1 ± 10.8, *p* < 0.05; Figure S2), while the UreI level was below the limit of detection. Moreover, to test the adherence ability of *H. pylori* to the GES-1 cells, *H. pylori* cells with/without daphnetin exposure were labeled with fluorescein-isothiocyanate (FITC) and analyzed by confocal microscopy. We demonstrated that daphnetin exposure significantly decreased the adherence of *H. pylori* to the GES-1 cells vs. control group (Figure 5C). These results suggested that daphnetin may prevent *H. pylori* colonization in human stomach.

### 2.6. The Cytotoxic Effect of Daphnetin on GES-1

The cytotoxic effect of daphnetin to GES-1 cells in medium (with/without serum) was investigated by using cell counting kit-8 (CCK-8) assay. The results showed that sub-MIC of daphnetin was well tolerated by GES-1 cells and there was no significant cytotoxic difference under both conditions (Table 3).

## 3. Discussion

The increasing prevalence of MDR *H. pylori* around the world has become one of the major causes of treatment failure in *H. pylori* infections [18,19]. The European Committee on Antimicrobial Susceptibility Testing (EUCAST) resistance breakpoints of clarithromycin and metronidazole to *H. pylori* were >0.5 μg/mL f and >8 μg/mL, respectively [20]. The global *H. pylori* antibiotic resistance rates were 17.2% for clarithromycin and 26.7% for metronidazole. In general, the resistance rate in developing countries is higher than that in developed countries [7,19]. Of note, metronidazole resistance rate of *H. pylori* isolated in the southeast coastal region of China is close to 100% according to a recent report [21]. In our study, the resistance rates for clarithromycin and metronidazole were much higher that the global *H. pylori* antibiotic resistance rates. According to the study from De Francesco et al., the clarithromycin- and metronidazole-resistance levels can be further classified into low level resistance (MICs from > 0.5 to ≤ 8 μg/mL for clarithromycin and from > 8 to ≤ 32 μg/mL for metronidazole) and high level resistance (MICs from > 8 to 256 μg/mL for clarithromycin and from > 32 to 256 μg/mL for metronidazole) [22]. All the 20 clinical strains in our study were low level resistance to clarithromycin and 50% (10/20) of clinical strains were high level resistance to metronidazole (See Table S1). The similar result was reported by Bai et al., indicating that antibiotic resistance in Chinese patients (MIC_50_ = 0.38 μg/mL for clarithromycin, and MIC_50_ = 128 μg/mL for metronidazole) [23]. Our study demonstrated that daphnetin has anti-*H. pylori* activity, with MICs ranging from 25 to 100 μg/mL, regardless of their resistance patterns to other antibiotics. Daphnetin was also reported to have antibacterial activity against other bacterial species, including *S. aureus*, *E. coli*, *P. aeruginosa*, and *R. solanacearum* [24,25]. However, the mechanism of daphnetin against bacteria has not been well studied.

Apoptosis was always occurred when stimulated by appropriate trigger in both eukaryotic multicellular organisms and in prokaryotic cells [26,27]. The cell morphology of apoptosis includes morphological transition from spiral to coccoid, increased in electrondense bodies, appear vacuoles [26,27]. Our morphological data were consistent with results of Cellini et al., who demonstrated that *H. pylori* cells change from typical spiral morphology to coccoid form as a response to environmental stress [28,29]. In addition, these phenomena were also similar with results of Shu et al., who observed a reduction in size and empty bubble degeneration in the daphnetin treatment group in synovial cells [30].

A stereotypical set of biochemical hallmarks of apoptosis (e.g., PS translocation, membrane depolarization, and DNA damage) have been proved in both eukaryotic and prokaryotic organisms [17,27,31,32]. In our experiments, we observed PS translocation and DNA damage significantly increased after daphnetin exposure as compared to control group. It is well known that DNA damage and membrane structure changes are the specific characteristics of apoptosis [33]. These phenotypes were also confirmed by other research groups studying prokaryotic organisms during apoptosis [17,28]. Although we could not detect one of the phenotypes related to apoptosis (e.g., membrane depolarization) in the current study [17]. The outcome may be related to decreasing reactive oxygen species (ROS) formation by daphnetin exposure (daphnetin-treated vs. control: 73.8 ± 7.11% vs. 10.42 ± 2.42%), due to there is a positive interaction between ROS accumulation and depolarization [34]. In addition, RecA plays a central role in the exhibition of these phenotypes [17], and we actually observed its expression significantly increased after daphnetin exposure. Therefore, our observations suggested that daphnetin exposure could induce a non-typical apoptosis in *H. pylori*.

RecA not only mediates cell death, but also plays an important role during stomach colonization [35]. For instance, RecA negatively regulates colonization-related *babA* gene expression [35]. In our current studies, we observed *recA* gene expression and RecA protein production were significantly increased by daphnetin exposure as compared to control. BabA is the best-characterized adhesion protein in *H. pylori*, which contributes the bacterium to attach to the glycosylated gastric epithelial cells [36]. Inconsistent with RecA’s function on *babA* expression, we found that the transcription of *babA* and BabA protein production were significantly decreased with daphnetin treatment vs. controls. UreI is a proton-gated urea channel and plays an important role in *H. pylori* colonization on acidic stomach surface [37]. We found that *urel* gene was significantly decreased with daphnetin exposure vs. control group by qRT-PCR. However, we could not detect UreI protein expression in our study, which might be related to the limitation of our LC–MS/MS analyses, as well as the solubility of UreI protein [37]. As *babA* and *urel* play important roles in cell colonization, the decreased expression of these two genes may lead to decreased adherence of *H. pylori* to cells. Consistently, decreased adherence of *H.* pylori to GES-1 cells was observed with daphnetin exposure. In addition, we found no significant cytotoxicity of daphnetin to the GES-1 cell line, which is in agreement with published data [14,16]. These results suggested that daphnetin may have ability to prevent *H. pylori* colonization on the stomach.

## 4. Materials and Methods

### 4.1. Bacterial Strains and Materials

Twenty *H. pylori* strains from CAMS Collection Center of Pathogen Microorganisms (CCPM) were isolated from gastric antrum in Beijing, China (see Table S1). *H. pylori* ATCC43504 was a standard strain isolated from human gastric antrum in Australia. It is a metronidazole-resistant strain, while sensitive to other clinical antibiotics (e.g., clarithromycin). For the isolation of *H. pylori* strains, gastric mucosal specimens were collected, inoculated on agar plates containing 5% defibrinated sheep blood, and cultured at 37 °C under microaerobic conditions (10% CO_2_, 5% O_2_, 85% N_2_) for 3 days [38]. The isolated *H. pylori* strains were confirmed by standard biochemical tests (urease, catalase), 16S rRNA sequencing, and morphological analyses. The study *H. pylori* strains were frozen (BHI media with 30% glycerine) in cryobank tubes at −80 °C. Clarithromycin and metronidazole were purchased from National Institutes for Food and Drug Control, Beijing, China. Vancomycin was purchased from INALCO SPA in Milano, Lombardia, Italy. Trimethroprim, polymyxin B sulfate, amphotericin B, and cefsulodin sodium salt were purchased from Sangon Biotech Co., Ltd., Shanghai, China. Daphnetin was purchased from Meilun Biotech Co., Ltd., Dalian, China. β-cyclodextrin, fluorescein-isothiocyanate (FITC) and human serum albumin (HSA) were purchased from Sigma, St. Louis, MO, USA. DMEM and FBS were obtained from Gibco, Waltham, MA, USA.

### 4.2. Cell Cultures

Agar-based culture of *H. pylori*: Frozen *H. pylori* strains were revitalized and maintained on Columbia blood agar plates containing selective antibiotics (e.g., vancomycin, trimethroprim, polymyxin B sulfate, amphotericin B, and cefsulodin sodium salt), and cultured at 37 °C under microaerobic conditions (10% CO_2_, 5% O_2_, 85% N_2_) for 3 days [38].

Liquid broth-based culture of *H. pylori*: *H. pylori* cells were swap from agar plate, resuspended in Brucella broth containing 10% FBS and 1% vancomycin, and cultured at 37 °C under microaerobic conditions (10% CO_2_, 5% O_2_, 85% N_2_) for 3 days [38].

GES-1, an immortalized human gastric epithelial cell line was cultured in DMEM medium supplemented with 10% FBS in a humidified incubator [39].

### 4.3. MICs of Daphnetin, Metronidazole, and Clarithromycin on H. pylori Strains

The antibacterial activities of daphnetin, metronidazole and clarithromycin against *H. pylori* were examined by standard agar dilution test (CLSI [M45]). Briefly, a saline suspension equivalent to a 2.0 McFarland standard (about 10^8^ CFU/mL) was prepared from a Mueller-Hinton agar plate plus selective antibiotics [38]. The inoculum is replicated directly onto the antimicrobial agent-containing agar dilution plates (daphnetin: 3.125–400 μg/mL; metronidazole: 1–512 μg/mL; clarithromycin: 0.015–8 μg/mL). The plates were incubated at 37 °C for 3 days. *H. pylori* ATCC43504 strain was used as a control. The MIC was determined as the lowest concentration of drug showing no growth, a haze, one discrete colony, or multiple tiny colonies [40].

### 4.4. H. pylori Morphology Analyses by SEM and TEM

The morphology of *H. pylori* with/without daphnetin exposure was performed by SEM and TEM as previously reported with some modifications [41,42]. For SEM, *H. pylori* strains were incubated with/without 6.25 or 12.5 μg/mL daphnetin for 3 days, then collected and fixed with 2.5% glutaraldehyde. Postfixing, the samples were centrifuged to remove glutaraldehyde and resuspended in phosphate buffer. The bacterial suspensions were spotted on a polished silicon wafer and allowed to dry overnight in a biosafety cabinet. The samples were then coated with chromium before SEM imaging. For TEM, *H. pylori* cells were exposed with/without 12.5 μg/mL of daphnetin for 3 days, then collected and fixed with 2.5% glutaraldehyde at least 2 h at 4 °C. The fixed organisms were washed and postfixed with 1% osmium tetroxide. Then the samples were washed, dehydrated in a graded series of ethanol and embedded in Epon Araldite. Ultrathin sections containing the cells were placed on copper grids, stained with uranyl acetate and lead citrate, observed, and photographed with a TEM microscope (Hitachi, Tokyo, Japan).

### 4.5. Detection of Membrane Changes

*H. pylori* cells were grown as described in the ‘Cell culture’ section above. Briefly, *H. pylori* cells were scraped from the Mueller-Hinton agar plates with or without daphnetin. To monitor the degree of cell membrane structural changes [17], a TransDetect Annexin V-FITC/PI Cell Apoptosis Detection Kit (Transgen Biotech, Beijing, China) was used. For membrane depolarization experiment [17], staining of cells were performed using DiBAC (Invitrogen, Waltham, MA, USA). To determine the integrity of cell membrane, a bicinchoninic acid (BCA) protein assay kit was used. Briefly, *H. pylori* cells (0.5 McFarland) were cultured with or without daphnetin in Brucella broth for 24 h. The samples were centrifuged at 4 °C, the supernatants were treated with BCA assay reagent, and OD at 595 nm was measured [43].

### 4.6. Detection of DNA Damage

To detect DNA damage in *H. pylori* [44], a TransDetect In Situ Fluorescein TUNEL Cell Apoptosis Detection Kit (Transgen Biotech, Beijing, China) was employed. Accuri C6 (BD, Franklin Lakes, Germany) flow cytometer and LSM510 confocal (Zeiss, Oberkochen, Germany) were used to detect the fluorescence signal changes. All flow cytometry data were collected using the Accuri C6 software. At least 10,000 cells were collected and analyzed for each sample. Flow data were processed and analyzed with CFlow Plus (BD, Franklin Lakes, Germany).

### 4.7. RNA Isolation and Quantitative Real-Time PCR

Briefly, *H. pylori* cells were incubated with/without 12.5 μg/mL daphnetin for 3 days, then collected. Total RNA was isolated using an RNAprep pure Cell/Bacteria Kit (TianGen Biotech, Beijing, China). Primers used in this study are listed in Table 4. Primer Premier 5 was used to design the primers, and nucleotide search was performed based on *Helicobacter pylori* strain 26695 chromosome locus (HP0071 for *ureI*; HP1243 for *babA*; and HP0153 for *recA*). A housekeeping gene 16S rRNA was used as control [45]. qRT-PCR was performed on the 7500 fast using an SYBR Green Supermix, Life Technologies (AB & Invitrogen, Waltham, MA, USA). All assays were carried out at least in three independent experiments in triplicates. Relative quantification was calculated by the ΔΔ*C*t method.

### 4.8. Membrane Preparation and Proteomics by Liquid Chromatography–Mass Spectrometry/Mass Spectrometry Analyses

The membrane fraction of *H. pylori* was prepared as described previously with modifications [46,47]. In brief, *H. pylori* cells were harvested, washed in 20mM Tris-HCl (pH 7.5), and pelleted twice by centrifugation (4000× *g* for 5 min). Bacterial cells were suspended in 20 mM Tris-HCl (pH 7.5) and broken by repeated ultrasonication. The mixture was incubated at room temperature for 30 min. Cell debris were removed by centrifugation (9000× *g* for 20 min, 4 °C). Total membrane pellet was collected by centrifugation (50,000× *g* for 20 min, 4 °C), then resuspended in 20 mM Tris-HCl (pH 7.5) containing 2.0% (*w*/*v*) sodium lauryl sarcosine.

For shotgun proteomics [48], proteins were reduced by dithiothreitol at 95 °C for 5 min and alkylated with iodoacetamide in dark for 1 h. Proteins were digested by sequencing grade modified trypsin (1:50 *w*/*w*) overnight at 37 °C. Lastly, the sample was desalted by C18 reverse-phase ZipTip. Standard shotgun proteomics techniques [48,49] were used to analyze the samples on a Thermo Scientific Orbitrap Fusion Lumos equipped with a Thermo Scientific™ Nanospray Flex Ion Source and nano-LC 1200 (Thermo Fisher Scientific, Bremen, Germany). Briefly, protein digests were enriched on a trap column (Thermo Scientific™ Acclaim™ PepMap™ 100 C18 LC Column 164946 (75 μm × 20 mm)) and separated with another column (Thermo Scientific™ Acclaim™ PepMap™ 100 C18 LC Column 164943 (0.050 mm × 150 mm)). After sample loading, the gradient started from 2 to 8% of solvent buffer (acetonitrile with 0.1% formic acid) for 1 min and then from 8 to 30% of solvent buffer for 69 min. Then, the gradient quickly changed from 30 to 40% of solvent buffer for 14 min and from 40 to 100% of solvent buffer for 1 min. In the final stage, the mobile phase was kept at 100% of solvent buffer for 5 min. The eluted peptides were ionized online by electrospray ionization and transferred into an Orbitrap Fusion Lumos mass spectrometer which was operated in the positive mode to measure full scan Mass Spectrometry (MS) spectra (from *m*/*z* 350–1550 in the Orbitrap analyzer at resolution *R* = 120,000 (MS1) and 15,000 (MS2). Higher-energy C-trap dissociation collision Energy was 32%.

For database analyses, unbiased data-dependent MS/MS acquisition was employed in peptide/protein identification. These initial data-dependent runs were searched against *H. pylori* ATCC 43504 and ATCC 26695 databases. Thermo Scientific™ Proteome Discoverer™ version 2.2 was used to analyze the quantitative data. The search parameters were set to MS accuracy 10 ppm, MS/MS accuracy 0.02 Da, dynamic modification (protein terminus) for acetyl, dynamic modification for oxidation, and static modification for carbamidomethyl.

### 4.9. H. pylori Adhesion Assays

To test the effect of daphnetin on *H. pylori* adherence to GES-1 cells, an adhesion assay was performed as described previously with minor modifications [50]. Briefly, GES-1 cells were seeded on cover glass bottom dishes and cultured at 37 °C with 5% CO_2_, until appropriate confluence (80–90%) was reached. Samples were then infected with FITC-labeled *H. pylori* (with or without daphnetin exposure). For *H. pylori* FITC-labeling, a previously described method [51] with minor modifications was used. Briefly, after 3 days incubation, *H. pylori* cells were harvested from agar plates with/without daphnetin exposure, and resuspended in 1.0 mL of 0.15 M NaCl and 0.1 M Na_2_CO_3_, pH 9.0 in double-distilled water by gentle pipetting. *H. pylori* cells were adjusted to 1.0 McFarland. Ten microliters of freshly prepared 1% FITC in dimethyl sulfoxide (DMSO) were added to the suspension, then incubated for 1 h at room temperature in the dark. Bacteria were recovered by centrifugation at 3000× *g* for 5 min, resuspended by gentle pipetting in 1.0 mL PBS supplemented with 5% inactivated fetal bovine serum, 0.2% BSA and 0.05% Tween 20. Add the FITC-labeled *H. pylori* cells into the dishes and incubated 4 h at 37 °C. After incubation, three washes were performed with PBS to remove nonadherent bacteria. LSM710 confocal was used to observe *H. pylori* adherence to GES-1 cells (Zeiss, Germany).

### 4.10. Cell Cytotoxicity Assays

Cell cytotoxicity was tested by the CCK-8 assay [52]. Briefly, GES-1 cells were plated in a 96-well plate. After overnight incubation, the medium were replaced by with/without serum medium, and then different concentrations of daphnetin were added. After 24 h of incubation, the cells were treated with CCK-8 assay reagent, and OD at 450 nm was measured.

### 4.11. Statistical Analyses

Descriptive statistics of samples in the detection of the cell-related changes were presented as means and SD from at least two independent experiments. Comparisons between control and daphnetin-treated groups were performed via unpaired 2-tailed Student’s *t*-test. *p* < 0.05 was considered statistically significant.

## 5. Conclusions

**Figure 6:**
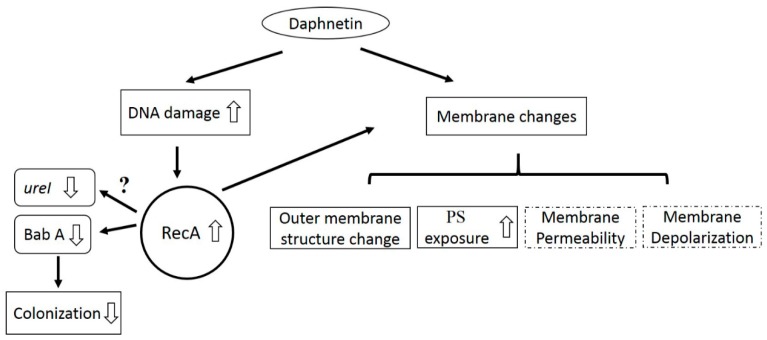
Hypothesized model of the mechanism(s) of daphnetin against *H. pylori.* Daphnetin exposure caused DNA damage and subsequently induced *recA* expression. In addition, *recA* negatively regulated *babA* transcription. To our best knowledge, no study indicated a direct interaction between *recA* and *urel*. Lower *babA* and *urel* transcription, and their respective protein production could reduce *H. pylori* adherence to GES-1 cells. Moreover, daphnetin exhibited effect on membrane changes (e.g., outer membrane structural change and increased PS exposure). In the current study, no significant impact of daphnetin on membrane permeability and depolarization was observed (dotted line indicates no statistical significance between control and daphnetin exposure groups).

In conclusion, the anti-*H. pylori* activity of daphnetin and relevant mechanisms of its action were reported in the current study. Daphnetin exhibited anti-MDR *H. pylori* activities. The mechanisms of its action attributed to induce membrane structure changes, DNA damage, and increase RecA expression. In addition, daphnetin exposure resulted with decreased colonization related gene expression (e.g., *babA* and *urel*) and adherence to GES-1 cells with no significant cytotoxicity to the cell line (Figure 6). Taken together, these results suggested that daphnetin has a potential to be an effective anti-*H. pylori* agent. Future studies, including in vivo anti-*H. pylori* activity evaluation and synthesis of daphnetin-derivatives with better biological activity, are expected.

## Figures and Tables

**Table 1:** Minimum inhibitory concentrations (MICs) of daphnetin, metronidazole, and clarithromycin against *H. pylori* strains.

Antibiotics	MICs (μg/mL)		Percent of Resistance (%) ^b^
	20 Clinical Isolates	ATCC43504 ^a^	
Daphnetin	25–100	25	NA ^c^
Clarithromycin	0.016–4	0.016	25%
Metronidazole	4–256	128	85%

^a^*H. pylori* ATCC43504 strain served as MIC quality control (metronidazole: 64–256 μg/mL; clarithromycin: 0.015–0.12 μg/mL). ^b^ Metronidazole: ≤ 8 μg/mL for susceptible and > 8 μg/mL for resistant; clarithromycin: ≤ 0.25 μg/mL for susceptible, 0.5 μg/mL for intermediate and > 0.5 μg/mL for resistant. ^c^ NA: not applicable.

**Table 2:** Membrane changes induced by daphnetin.

Groups	Mean of the Positive Fluorescence ± SD (%)			Protein Leakage (μg/mL)
	PS Translocation	Membrane Permeability	Membrane Depolarization	
Control	5.93 ± 1.25	7.78 ± 0.62	9.26 ± 1.34	0.56 ± 0.01
Daphnetin (12.5 μg/mL)	56.99 ± 5.78 *	5.06 ± 3.40	8.87 ± 2.71	0.60 ± 0.03

* *P* < 0.001 vs. control.

**Table 3:** The cytotoxic effects of daphnetin to GES-1 cells in medium (with/without serum).

Groups	Viability (mean ± SD/%) ^a^	
	DMEM	DMEM + 10% FBS ^b^
Control	100.00 ± 7.79	100.00 ± 2.30
Daphnetin 12.5 μg/mL	84.43 ± 5.95	81.14 ± 11.52

^a^: The viability of control group without daphnetin exposure was normalized as 100%. ^b^: DMEM: Dulbecco’s modified Eagle’s medium; FBS: fetal bovine serum.

**Table 4:** The qRT-PCR primers used in this study.

Primers	Sequence
*ureI*	Forward: CCCCTGTAGAAGGTGCTGAA Reverse: GCCGCATACAAGTAGGTGAAAC
*babA*	Forward: AAGCCTATCAAATCCTCCAAACG Reverse: TGGCGAGCAGTTATTATTCCCT
*recA*	Forward: CTAAGAGGTTGGGCGTGGA Reverse: CAATCCCTCCGCTTCTGGT
16s rRNA	Forward: GTGCCAGCMGCCGCGGTAA Reverse: GACTACHVGGGTATCTAATCC

## Supplementary Materials

Supplementary materials can be found at http://www.mdpi.com/1422-0067/20/4/850/s1. Table S1. MICs of metronidazole, clarithromycin and daphnetin against *H. pylori* strains. Figure S1. The expression of RecA in *H. pylori* with/without daphnetin exposure. Figure S2. The expression of BabA in *H. pylori* with/without daphnetin exposure.
