# Supplementary material for: Daphnetin: A Novel Anti-Helicobacter pylori Agent
Source: Int J Mol Sci. 2019 Feb 15;20(4):850. doi: 10.3390/ijms20040850 (PMC6412720; doi:10.3390/ijms20040850)
Supplement: Supplementary file 1 [file ijms-20-00850-s001.pdf]

International Journal of
Molecular Sciences

MDPI

Table S1. MICs of metronidazole, clarithromycin and daphnetin against *H. pylori* strains.

Strain Number	Source	Specimen Date	Location	MICs (μg/ml)		
				Daphnetin<br>NA [a]	Clarithromycin<br>S≤0.25, R>0.5	Metronidazole<br>S≤8, R>8
CCPMAP 150001	gastric antrum	2015	CCPM [a]	25	0.016	32
CCPMAP 150002	gastric antrum	2015	CCPM	50	1	2
CCPMAP 150003	gastric antrum	2015	CCPM	50	0.016	64
CCPMAP 160001	gastric antrum	2016	CCPM	50	0.016	32
CCPMAP 160002	gastric antrum	2016	CCPM	50	0.032	32
CCPMAP 160003	gastric antrum	2016	CCPM	25	0.016	128
CCPMAP 160004	gastric antrum	2016	CCPM	25	0.064	64
CCPMAP 160005	gastric antrum	2016	CCPM	50	0.016	128
CCPMAP 160006	gastric antrum	2016	CCPM	25	0.064	64
CCPMAP 160007	gastric antrum	2016	CCPM	100	0.032	32
CCPMAP 160008	gastric antrum	2016	CCPM	50	0.016	32
CCPMAP 160009	gastric antrum	2016	CCPM	50	0.016	64
CCPMAP 160010	gastric antrum	2016	CCPM	100	0.032	16
CCPMAP 160011	gastric antrum	2016	CCPM	50	2	32
CCPMAP 160012	gastric antrum	2016	CCPM	50	0.016	64
CCPMAP 160013	gastric antrum	2016	CCPM	50	0.032	16
CCPMAP 160014	gastric antrum	2016	CCPM	50	4	4
CCPMAP 160015	gastric antrum	2016	CCPM	100	0.064	128

CCPMAP 160016	gastric antrum	2016	CCPM	100	4	2
CCPMAP 160017	gastric antrum	2016	CCPM	100	4	64
ATCC43504	gastric antrum	2011	ATCC [a]	25	0.016	128

[a]: CCPM, CAMS Collection Center of Pathogen Microorganisms; NA, not applicable; ATCC, the American Type Culture Collection.

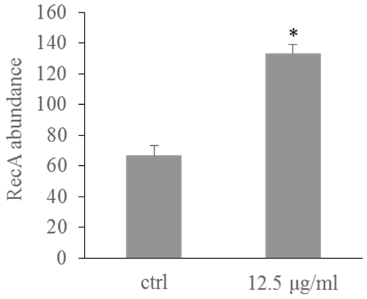

Figure S1. The expression of RecA in *H. pylori* with/without daphnetin exposure. Data was presented as mean and standard deviations (SD). * $P < 0.001$ vs. control.

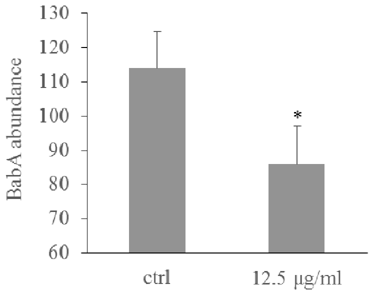

Figure S2. The expression of BabA in *H. pylori* with/without daphnetin exposure. Data was presented as mean and standard deviations (SD). * $P < 0.05$ vs. control.
